# *Panzerina lanata* accelerates methicillin-resistant *Staphylococcus aureus* eradication by promoting migration and activation of neutrophils

**DOI:** 10.3389/fphar.2024.1501744

**Published:** 2025-01-14

**Authors:** Shuai Dong, Xingyuan Bai, Bin Chen, Minzhe Fan, Qi Liu, Yubo Zhao, Linsen Li, Dan Zhu

**Affiliations:** Laboratory of Pharmacology, Key Laboratory of Ethnomedicine of Ministry of Education, School of Pharmacy, Minzu University of China, Beijing, China

**Keywords:** *Panzerina lanata*, methicillin-resistant *Staphylococcus aureus*, migration, activation, neutrophil

## Abstract

**Background:**

*Panzerina lanata* (Lanata) is generally used to treat pustule infection in Inner Mongolia folk medicine and is called “the holy medicine for pustule.” However, the pharmacological mechanism of Lanata in treating pustule infection is still unclear.

**Aims:**

This study aimed to investigate the therapeutic effects of Lanata on skin infection and explore the underlying mechanisms.

**Methods:**

A skin wound methicillin-resistant *Staphylococcus aureus* (MRSA) infection mouse model was established to evaluate the healing effect of Lanata on infected wounds. *In vitro* assays were also conducted to determine the antibacterial activity of Lanata. Flow cytometry and immunohistochemistry were used to dynamically detect the number of neutrophils in the bone marrow, peripheral blood, and MRSA-infected wound. Protein expression in the infected wound skin was detected by a protein chip. Using an air pouch MRSA infection mouse model, the number of neutrophils, reactive oxygen species (ROS) level in neutrophils, and neutrophil extracellular trap (NET) formation were dynamically detected by flow cytometry and immunofluorescence. RNA-seq, RT-qPCR, flow cytometry, ELISA, and CXC chemokine receptor 2 (CXCR2) and P-selectin glycoprotein ligand-1 (PSGL-1) inhibitors were used to explore the mechanism of Lanata in regulating neutrophils.

**Results:**

*In vitro* assays showed that Lanata had no direct antibacterial activity. In skin wound MRSA-infected mouse, Lanata promoted the rapid migration of neutrophils from the bone marrow via peripheral blood to the wound site to eradicate MRSA in the acute stage of infection and accelerate wound healing. Skin protein chip analysis showed that Lanata upregulated CXCR2 and PSGL-1 protein levels in skin wounds. Furthermore, analysis using the air pouch MRSA infection mouse model found that Lanata not only promoted the rapid migration of neutrophils from peripheral blood to the air pouch but also enhanced the activation of neutrophils, including the increase of ROS and the release of NETs, and upregulated the expression of CXCR2, PSGL-1, and myeloperoxidase (MPO) in neutrophils. Inhibition of CXCR2 and MPO significantly attenuated the effect of Lanata on promoting migration and activation of neutrophils.

**Conclusion:**

*Panzerina lanata* resists MRSA infection by promoting migration and activation of neutrophils to rapidly eradicate MRSA.

## 1 Introduction

Bacterial infectious diseases are highly prevalent and are an important cause of death worldwide. An estimated 7.7 million infection-related deaths were reported to be linked to 33 bacterial pathogens worldwide in 2019 (Collaborators, 2022). Skin and soft tissue infection is one of the most common bacterial infections, and the rapid spread of methicillin-resistant *Staphylococcus aureus* (MRSA) strains contributes to its increasing incidence (Watkins and David, 2021). With the widespread use of antibiotics, multi-drug resistant bacteria continue to emerge, and the treatment of bacterial infectious diseases is facing major challenges. This forces medical workers to open up new ideas and ways to find anti-infective drugs.

*Panzerina lanata* (Lanata) is a plant of the Lamiaceae family and genus *Panzerina*, and its aboveground part is the characteristic Mongolian medicinal herb called Baiyimucao in China. Lanata is generally used to treat pustule infection in Inner Mongolia folk medicine and is called “the holy medicine of pustule” (Wu and Fu, 1988; Xing, 2006; Meng, 2009). We have been studying the efficacy, toxicity, and chemical composition of Lanata since 2009 (Li, 2011; Zhou, 2022). Our previous research identified a total of 20 natural products in Lanata, including alkaloids, flavonoids, organic acids, and lignans (Mi et al., 2021). However, the pharmacological mechanism of Lanata in treating pustule infection is still unclear. In this study, we used MRSA as the source of infection to first evaluate the effect of Lanata on skin MRSA infection in mice. We found that Lanata clearly promoted healing of infected wounds, accompanied by a significant change in the number of neutrophils in peripheral blood. However, we also observed that Lanata had no direct antibacterial activity *in vitro*. Therefore, our study mainly focused on investigating neutrophils’ dynamics and activity in the context of infection.

Neutrophils are the key effector cells of the innate immune system, and being the professional phagocytes, they are the first one migrating to the site of infection, thus constituting the first line of defense (Rawat et al., 2023). Upon arrival at the site of infection, neutrophils eradicate bacteria through phagocytosis and the release of reactive oxygen species (ROS), degranulation, and neutrophil extracellular traps (NETs) (Cristinziano et al., 2022), which are crucial for the body’s ability to eradicate bacteria. Neutrophils are becoming a new target for the treatment of multi-inflammatory diseases (Cross et al., 2020).

In this study, we investigated the therapeutic effect of Lanata on skin infection and its underlying mechanisms, including migration and activation of neutrophils during MRSA infection.

## 2 Materials and methods

### 2.1 Materials and reagents

The following materials and reagents were purchased: MRSA strain ATCC43300 (General Microbiological Culture Collection Center, Beijing, China); 2,3,5-triphenyltetrazolium chloride (TTC) (Beijing Wanjing Lizhi I Biotechnology Ltd., Beijing, China); linezolid (Aladdin Industrial Corporation, Los Angeles, United States); SB225002 and 4-aminobenzoic hydrazide (ABAH) (TargetMol Chemicals Inc., Boston, United States); APC anti-mouse CD45 antibody (Clone: 30-F11), PE anti-mouse/human CD11b antibody (Clone: M1/70), PerCP/Cyanine5.5 anti-mouse Ly-6G antibody (Clone: 1A8), FITC anti-mouse CXCR2 antibody (Clone: SA044G4), and RBC lysis buffer (BioLegend Inc., San Diego, United States); Alexa Fluor 488 anti-mouse PSGL-1 antibody (Clone: 4RA10) (Thermo Fisher Scientific, Inc., New York, United States); rabbit monoclonal MPO antibody (Abcam Ltd., Cambridge, United Kingdom); rabbit neutrophil elastase antibody (Novus Biologicals Inc., Littleton, United States); Alexa Fluor 488 goat anti-rabbit IgG secondary antibody and DAPI (Wuhan Servicebio Technology Ltd., Wuhan, China); DCFH-DA (Beyotime Biotechnology Ltd., Shanghai, China); Mouse PSGL-1 and CXCR2 ELISA kits (Jingmei Biotechnology Ltd., Yancheng, China); MPO ELISA kit (Jiubang Science & Technology Ltd., Quanzhou, China); RIPA lysis buffer (Lablead Biotechnology Ltd., Beijing, China); and Mouse StarScript III RT Mix with gDNA Remover and 2 × RealStar Fast SYBR qPCR Mix (GenStar Biotech Ltd., Beijing, China).

### 2.2 Preparation of Lanata

The Lanata herb was collected from the Dalad Banner of Inner Mongolia Province, China, and identified by Professor Chun-Lin Long (College of Life and Environmental Sciences, Minzu University of China). The herb (10 kg) was reflux-extracted with 80 L 75% ethanol (1:8, w/v) for 2 h. The extracting liquid was transferred to the concentration tank; then, the herb remnant was reflux-extracted for 1 h. The two extraction solutions were concentrated under reduced pressure, and 1.25 kg extract was obtained. The extract rate was 12.5%. The extract was stored at −20°C and dissolved in distilled water to the indicated concentrations before use.

### 2.3 Experimental animals

Male ICR mice (23–25 g, 5 weeks old) were purchased from SPF (Beijing) Biotechnology Co., Ltd. (SCXK 2019-0010) and housed in a room with stable temperature (22°C ± 2°C), humidity (50% ± 10%), and 12 h/12 h light/dark cycle. All mice had access to free water and food and underwent one-week accommodation before experiments. All procedures were approved by the Biological and Medical Ethics Committee, Minzu University of China (approval no. ECMU2023001AO).

### 2.4 Skin wound MRSA infection

After the mice were anesthetized with 1% pentobarbital sodium (50 mg/kg), hair was removed from a 3 cm × 3 cm area on the back of anesthetized mice and a skin area of 5-mm-diameter area was cut. Then, 5 μL of the 2 × 10^8^ CFU/mL MRSA bacterial solution (0.9% saline) was added to the wound. Mice in the sham group were subjected to the same surgical procedures without MRSA.

The skin wound MRSA infection mice were divided into the following five groups: MRSA (model, vehicle), MRSA + Lanata-L (Lanata low-dose, 2 g/kg herb), MRSA + Lanata-M (Lanata medium-dose, 4 g/kg herb), MRSA + Lanata-H (Lanata high-dose, 8 g/kg herb), and MRSA + Linezolid (linezolid, 0.12 g/kg). All drugs with distilled water as the vehicle were administered by oral gavage immediately after infecting with MRSA. Mice in the sham and the control groups were given an equal volume of vehicle. Mice in all groups were administered five times (0 day, 1 day, 2 days, 3 days, and 4 days).

### 2.5 Air pouch MRSA infection

After the mice were anesthetized, 3 mL air sterilized through a 0.22-μM filter was injected subcutaneously on the dorsal region of anesthetized mice to generate an air pouch, which was then injected with 1 mL 1 × 10^6^ CFU/mL MRSA bacterial solution (0.9% saline) or 1 mL of 0.9% saline (control group).

The air pouch MRSA infection mice were divided into two groups: MRSA (model, vehicle) and MRSA + Lanata (Lanata, 4 g/kg herb). Lanata and distilled water as the vehicle were administered by oral gavage immediately after infecting with MRSA. Mice in the control group were given an equal volume of vehicle. Mice in all groups were administered once.

### 2.6 Healing rate of infected wounds

A metal foil with an inner diameter of 9 mm was placed over the wound as a reference for area measurement, and ImageJ software was used to calculate the area of photographed wounds. The healing rate of infected wounds was calculated according to the following formula: healing rate of infected wound = (wound area 0 day post-operation − wound area n days post-operation)/wound area 0 day post-operation × 100%.

### 2.7 Antibacterial activity of Lanata

The zone of growth inhibition test was performed according to previous reports (Moussa et al., 2013). The concentrated extract of Lanata was dissolved in the tryptic soy broth (TSB) liquid medium to a concentration of 200 mg/mL, and serial dilutions of Lanata (100, 50, 25, 12.5, and 6.25 mg/mL) were prepared in a 96-well plate in a maximum volume of 100 μL of TSB. The 100 μL 1 × 10^6^ CFU/mL MRSA bacterial solution was added to each well. Here, 200 μL TSB was used as negative control, while 100 μL of the TSB +100 μL 1 × 10^6^ CFU/mL MRSA bacterial solution was used as positive control. After incubation at 37°C for 24 h, 20 μL of the 0.5% TTC solution was added to each well, followed by incubation at 37°C for 24 h and OD measurement at 485 nm.

### 2.8 Detection of neutrophils in peripheral blood

Blood was collected from the orbital sinus of anesthetized mice at 2 h, 6 h, 12 h, 24 h, 48 h, and 72 h (skin wound) or at 4 h, 6 h, 12 h, and 24 h (air pouch) post-MRSA infection. A volume of 20 μL blood was added to 1 mL of 1 × red blood cell (RBC) lysis buffer and incubated at 37°C protected from light for 5 min, followed by centrifugation at 350 g/min for 5 min. The supernatant was discarded, and the cell pellet was washed with PBS and incubated with the APC anti-mouse CD45 antibody, PE anti-mouse CD11b antibody, and PerCP/Cyanine5.5 anti-mouse Ly-6G antibody. After washing with PBS, the cells were analyzed on a FACSCelesta (BD, America), and data were analyzed with FlowJo v10.

### 2.9 Detection of neutrophils in the bone marrow

Mice were sacrificed following blood collection, and hind limbs were separated. The bone marrow cavity was rinsed with 1 mL PBS for three times to obtain the bone marrow cells, which were analyzed by flow cytometry, as described above.

### 2.10 Detection of neutrophils in air pouch

At 4 h, 6 h, 12 h, and 24 h post-infection, 1 mL solution (PBS containing 5.4 mM EDTA-Na_2_) was injected into the air pouch for lavage, and the lavage fluid was collected and centrifuged at 500 g/min for 10 min. The supernatant was discarded, and the cells in the air pouch were collected and analyzed by flow cytometry, as described above.

### 2.11 Hematoxylin and eosin staining and immunohistochemistry

At 2 h, 6 h, 12 h, 24 h, 48 h, and 72 h post-infection, the mice were sacrificed and a 2-cm × 2-cm wound skin was obtained to perform H&E staining and immunohistochemistry, according to previous reports (Yuan et al., 2014).

### 2.12 Detection of bacterial load

At 6 h, 12 h, 24 h, and 48 h post-MRSA infection, skin samples of 2 cm × 2 cm around the wound were obtained and added to 1 mL of 0.9% saline and immediately homogenized. At 4 h, 6 h, 12 h, and 24 h post-infection, the lavage fluid in the air pouch was collected and serially diluted in saline.

Serially diluted solutions of skin homogenates and air pouch lavage fluids were spread on a TSA plate and incubated at 37°C for 24 h to conduct colony count.

### 2.13 Protein chip analysis of the wound skin

At 6 h post-infection, skin samples of 2 cm × 2 cm around the wound were obtained, and RIPA lysis buffer was added and homogenized immediately. Lysates were centrifuged at 12,000 g/min for 10 min and sent to RayBiotech Guangzhou Co., Ltd. to detect the concentration of 200 proteins. A *p*-value < 0.05 and fold change >2 or <0.5 were set as thresholds for significantly differentially expressed proteins (DEPs). Enrichment analysis of DEPs was conducted using the GO (http://www.geneontology.org/) and KEGG (http://www.kegg.jp/) databases.

### 2.14 Detection of ROS in neutrophils

At 4 h, 6 h, 12 h, and 24 h post-MRSA infection, cells in the air pouch were washed with PBS and incubated with the APC anti-mouse CD45 antibody, PE anti-mouse CD11b antibody, PerCP/Cyanine5.5 anti-mouse Ly-6G antibody, and DCFH-DA. After washing with PBS, the cells were analyzed by flow cytometry, as described above.

### 2.15 Immunofluorescence staining of NETs

At 4 h, 6 h, 12 h, and 24 h post-infection, 5 μL cell suspension was evenly distributed on a slide in an area of 1 cm^2^, blocked with 5% BSA overnight at 4°C and incubated with the rabbit anti-neutrophil elastase (NE) antibody for 4 h at room temperature, washed with PBS, and incubated with Alexa Fluor 488 goat anti-rabbit IgG secondary antibody for 1 h and DAPI for 15 min at room temperature. Images were captured with the FV3000 confocal system (Olympus, Japan).

### 2.16 RNA-seq analysis of neutrophils

At 6 h post-infection, neutrophils in the air pouch were collected in both the MRSA and MRSA + Lanata groups. Cells were washed with PBS, and total RNA was extracted and quantified. The sequencing library was sequenced on a NovaSeq 6000 platform by Beijing Qinglian Biotech Co., Ltd. Differential expression analysis was performed using the Love et al. (2024) R package. A *p*-value < 0.05 and fold change >2 or <0.5 were set as thresholds for significantly differentially expressed genes (DEGs). Enrichment analysis of DEGs was conducted using GO and KEGG databases.

### 2.17 RT-qPCR determination

Neutrophils in the air pouch and blood were collected at 4 h, 6 h, 12 h, and 24 h post-infection. According to the RNA extraction method, total mRNA was extracted with TRIzol, chloroform, isopropanol, ethanol, and other reagents. The total RNA concentration was measured using NanoDrop 2000 (Thermo Fisher Scientific, United States), and reverse transcription was performed with StarScript III RT Mix with gDNA Remover. Results were analyzed using 2 × RealStar Fast SYBR qPCR Mix on LightCycler^®^ 96. Primers used are presented in Supplementary Table S1.

### 2.18 ELISA determination

Skin samples of 2 cm × 2 cm around the wound were obtained at 6 h post-infection. At 4 h, 6 h, 12 h, and 24 h post-infection, neutrophils in the air pouch and blood were collected. P-selectin glycoprotein ligand-1 (PSGL-1) and CXC chemokine receptor 2 (CXCR2) in the skin and myeloperoxidase (MPO) in neutrophils were determined, following the manufacturer’s instructions.

### 2.19 Detection of CXCR2 and PSGL-1 in neutrophils

At 4 h, 6 h, 12 h, and 24 h post-infection, cells in peripheral blood and air pouch were washed with PBS and incubated with the APC anti-mouse CD45 antibody, PE anti-mouse CD11b antibody, PerCP/Cyanine5.5 anti-mouse Ly-6G antibody, and FITC anti-mouse CXCR2 antibody or Alexa Flour 488 (AF488) anti-mouse PSGL-1 antibody. After washing with PBS, the cells were analyzed on a FACSCelesta instrument, and data were analyzed with FlowJo v10.

### 2.20 Inhibition of MPO and CXCR2 in neutrophils

At 0.5 h before MRSA was injected into the air pouch, the CXCR2 inhibitor SB225002 (10 mg/kg) or MPO inhibitor ABAH (40 mg/kg) was intraperitoneally injected into mice. The air pouch MRSA infection mice were divided into the following six groups: the model group (MRSA, vehicle), MRSA + Lanata group (MRSA + Lanata, 4 g/kg herb), MRSA + SB225002 group (MRSA + SB225002, vehicle), MRSA + ABAH group (MRSA + ABAH, vehicle), MRSA + Lanata + SB225002 group (MRSA + Lanata + SB225002, 4 g/kg herb), and MRSA + Lanata + ABAH group (MRSA + Lanata + ABAH, 4 g/kg herb). At 4 h post-infection, immunofluorescence of NETs in the air pouch was performed. At 6 h post-infection, the number of neutrophils and ROS levels in neutrophils from the air pouch were determined.

### 2.21 Statistical analysis

Statistical analysis was conducted with SPSS 26.0. Comparisons between two groups were assessed using Student’s t-test. Comparisons for three or more groups were analyzed using one-way analysis of variance (ANOVA), followed by a least significant difference (LSD) *post hoc* test. All data were expressed as mean ± standard deviation (SD). *p* < 0.05 was considered to be statistically significant.

## 3 Results

### 3.1 Lanata promoted healing of MRSA-infected wounds without exerting direct antibacterial activity

**FIGURE 1:**
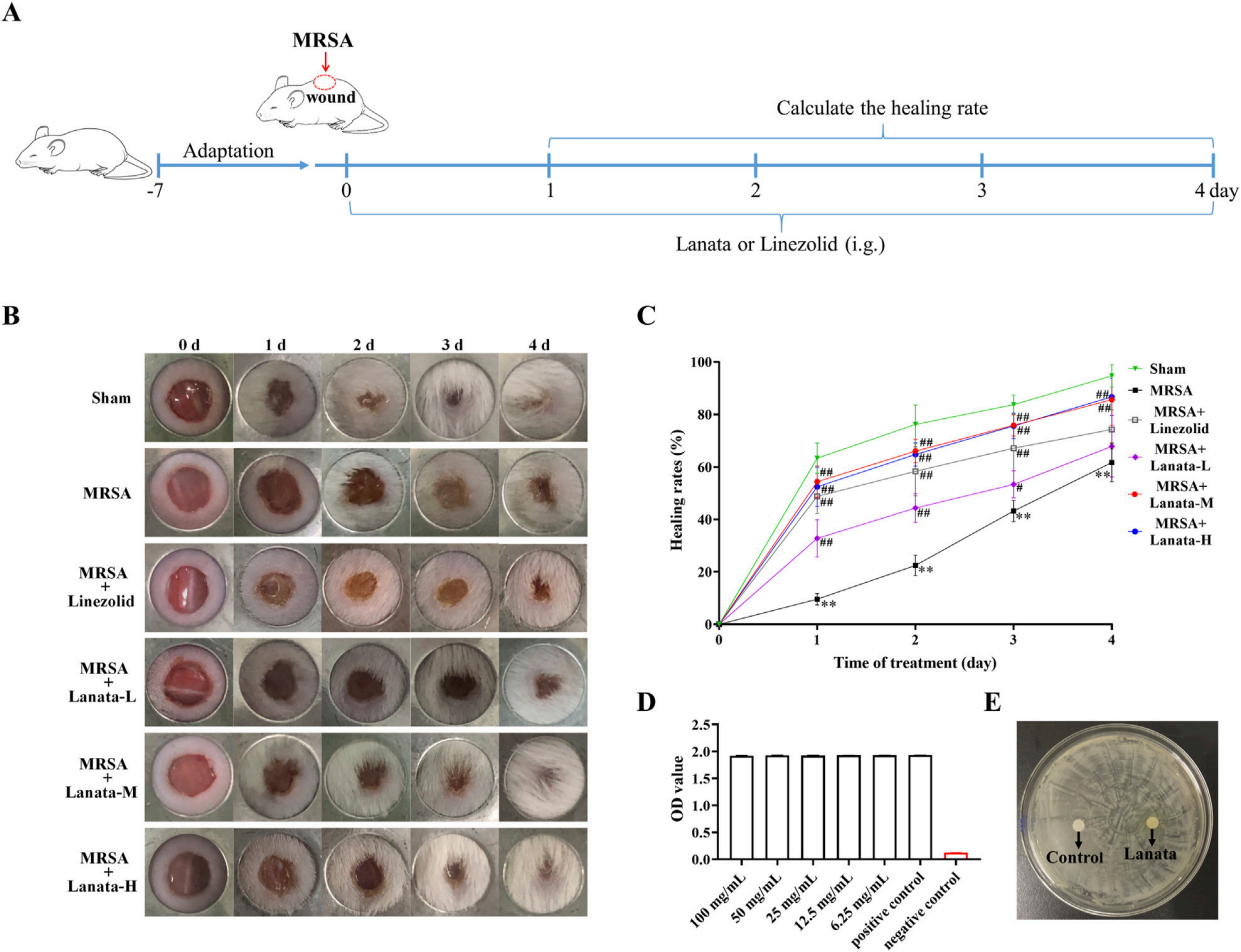
Lanata promoted healing of MRSA-infected wounds without direct antibacterial activity. **(A)** Experimental design. **(B, C)** Healing rates of MRSA infected wounds in each group of mice. **(D)** TCC assay and **(E)** zone of growth inhibition test were used to evaluate the antibacterial activity of Lanata. Data were presented as the mean ± SD, *n* = 6. **p < 0.01 vs. Sham group; ^#^ *p* < 0.05, ^##^ *p* < 0.01 vs. MRSA group.

Considering the therapeutic effect of Lanata on abscess infection, we established a skin wound MRSA infection mouse model to investigate the healing effect of Lanata on infected wounds (Figure 1A). Wound healing of mice in the Sham group was the fastest, being almost completely healed at 72 h post-infection (Figures 1B, C), while healing in the MRSA group was significantly slower due to bacterial infection. Compared with the MRSA group, the healing rate in MRSA + Linezolid and MRSA + Lanata groups increased in different degrees. The healing effect in the MRSA + Lanata-M and MRSA + Lanata-H groups was most prominent, with the largest difference observed at 24 h. However, Lanata had no direct antibacterial activity against MRSA *in vitro* (Figures 1D, E).

### 3.2 Lanata promoted migration of neutrophils to the infected wound and eradication of MRSA

**FIGURE 2:**
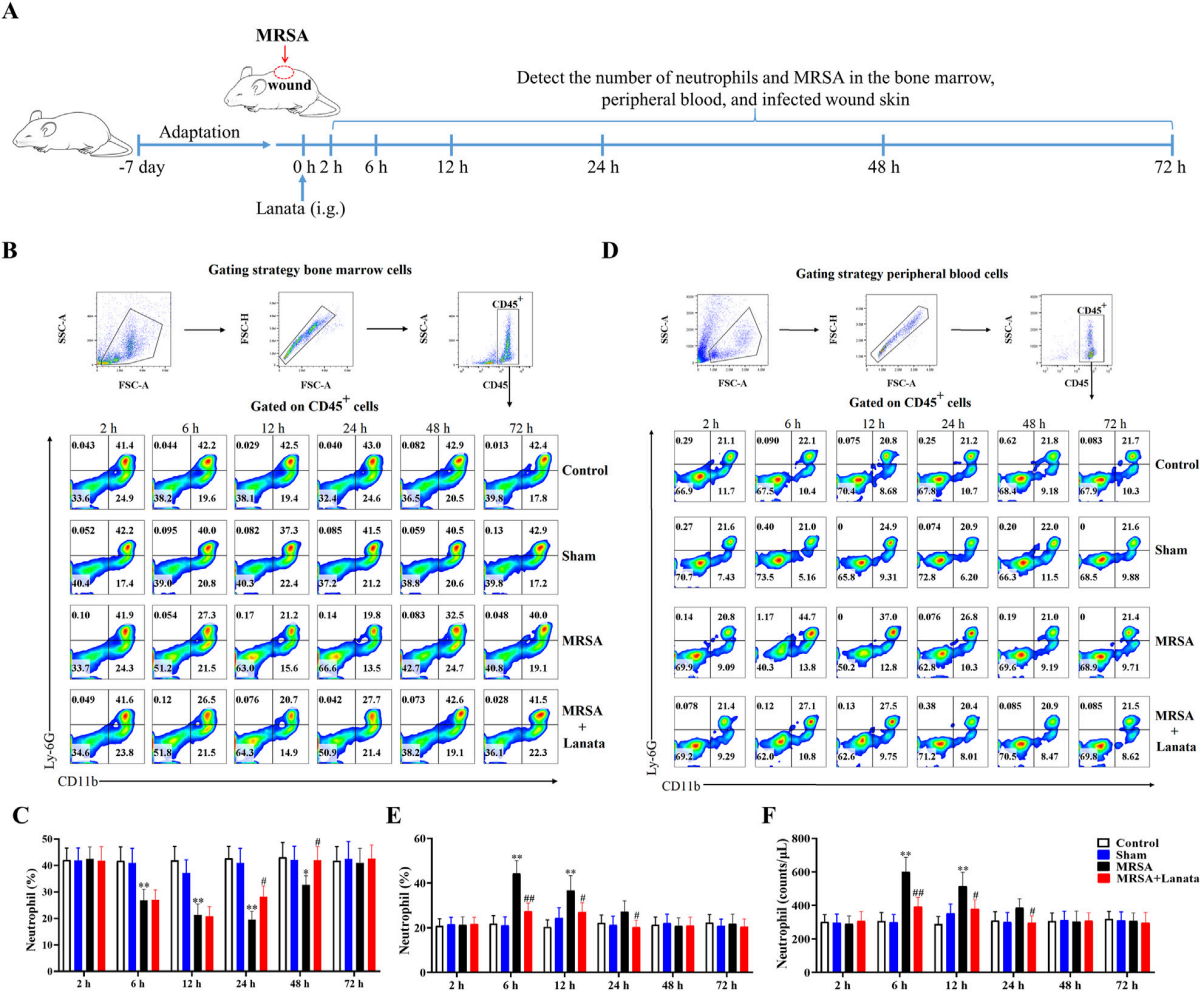
Lanata promoted migration of neutrophils to infected wound. **(A)** Experimental design. **(B)** Flow cytometry analysis of neutrophils in the bone marrow. **(C)** Percentage of neutrophils in bone marrow leukocytes. **(D)** Flow cytometry analysis of neutrophils in peripheral blood leukocytes. **(E)** Percentage and **(F)** number of neutrophils in peripheral blood leukocytes. The data are presented as the mean ± SD, *n* = 3. *p < 0.05, **p < 0.01 vs. Control group; ^#^ *p* < 0.05, ^##^ *p* < 0.01 vs. MRSA group.

**FIGURE 3:**
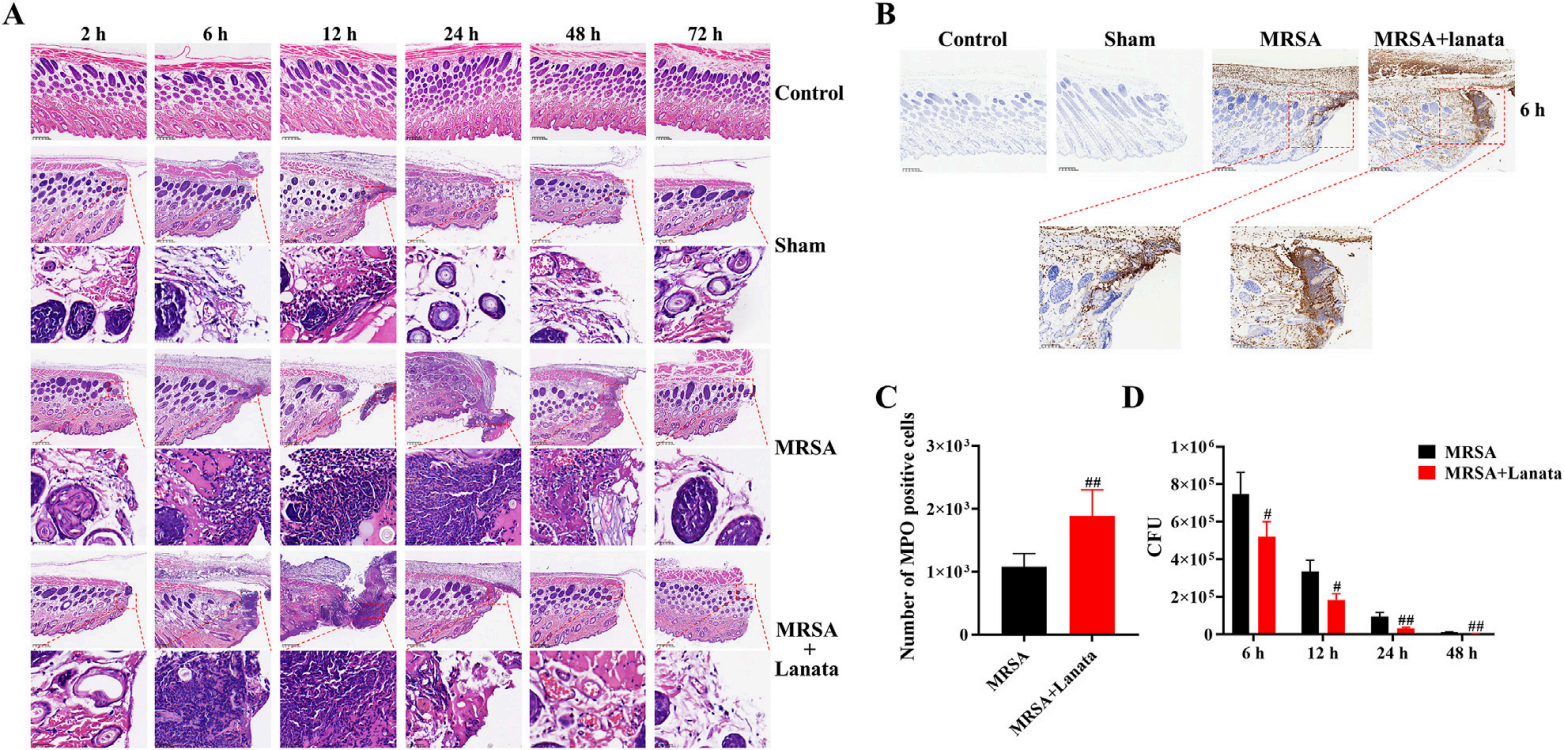
Neutrophil infiltration in the infected wound skin of mice. **(A)** H&E was used to observe neutrophil infiltration in the infected wound skin. **(B)** Immunohistochemistry of MPO expression was used to observe the number of neutrophils in the infected wound skin at 6 h post-infection. **(C)** The number of MPO-expressing positive cells in each group of mice was counted based on the location and area of the frame-selected skin-infected wounds in this figure **(B)**. **(D)** Number of MRSA in the infected wound skin. Data were presented as the mean ± SD, *n* = 3. ^#^ *p* < 0.05, ^##^ *p* < 0.01 vs. MRSA group.

We observed the spatiotemporal dynamics of neutrophil migration during MRSA infection to investigate whether mice treated with Lanata could resist MRSA infection by regulating neutrophils (Figure 2A). At 2 h post-infection, there was no neutrophil infiltration in the infected wound of mice in the MRSA and MRSA + Lanata groups (Figure 3A), and the proportion and number of neutrophils in the bone marrow and the peripheral blood remained homeostatic (Figures 2B–F).

At 6 h, a considerable number of neutrophils began to migrate into the infected wound in the MRSA group (Figures 3A–C) and the proportion of neutrophils in the bone marrow significantly decreased, while the number of neutrophils in the peripheral blood significantly increased (Figures 2B–F), indicating that neutrophils in the bone marrow had been rapidly mobilized into the peripheral blood. Compared with the MRSA group, the number of neutrophils in the infected wound in the MRSA + Lanata group significantly increased, while simultaneously, MRSA and the number of neutrophils in the peripheral blood significantly decreased (Figures 2F, 3A–D).

At 12 h, the number of neutrophils in the infected wound in the MRSA group significantly increased compared with 6 h (Figure 3A), while the proportion and number of neutrophils in the bone marrow and peripheral blood were significantly reduced (Figures 2B–F). Interestingly, the peak in the number of neutrophils in the infected wound in the MRSA + Lanata group was observed in the acute stage of infection, and MRSA and the number of neutrophils in the peripheral blood were significantly lower than those in the MRSA group (Figures 2F, 3A–D).

At 24 h, the number of neutrophils in the infected wound in the MRSA group reached the peak (Figure 3A), while the number of neutrophils in the bone marrow and peripheral blood further decreased (Figures 2B–F). Meanwhile, the number of neutrophils in the infected wound in the MRSA + Lanata group was significantly reduced compared with 12 h and that in the MRSA group, and MRSA was almost completely eradicated, and the number of neutrophils in the bone marrow and peripheral blood began to return to a steady state.

At 48 h, MRSA was almost completely eradicated in the MRSA group, the number of neutrophils was significantly reduced compared with 24 h (Figure 3A), and the number of neutrophils in the peripheral blood and the bone marrow began to return to the steady state (Figures 2B–F). In the MRSA + Lanata group, there was no obvious neutrophil infiltration in the infected wound, and the number of neutrophils in the bone marrow and peripheral blood had returned to a homeostatic state.

Together, the above results suggest that Lanata promoted the rapid migration of neutrophils from the peripheral blood to the infected wound to eradicate MRSA quickly in the acute stage of infection, thus promoting healing of the infected wound.

### 3.3 Lanata upregulated the protein levels of CXCR2 and PSGL-1 in the infected wound

**FIGURE 4:**
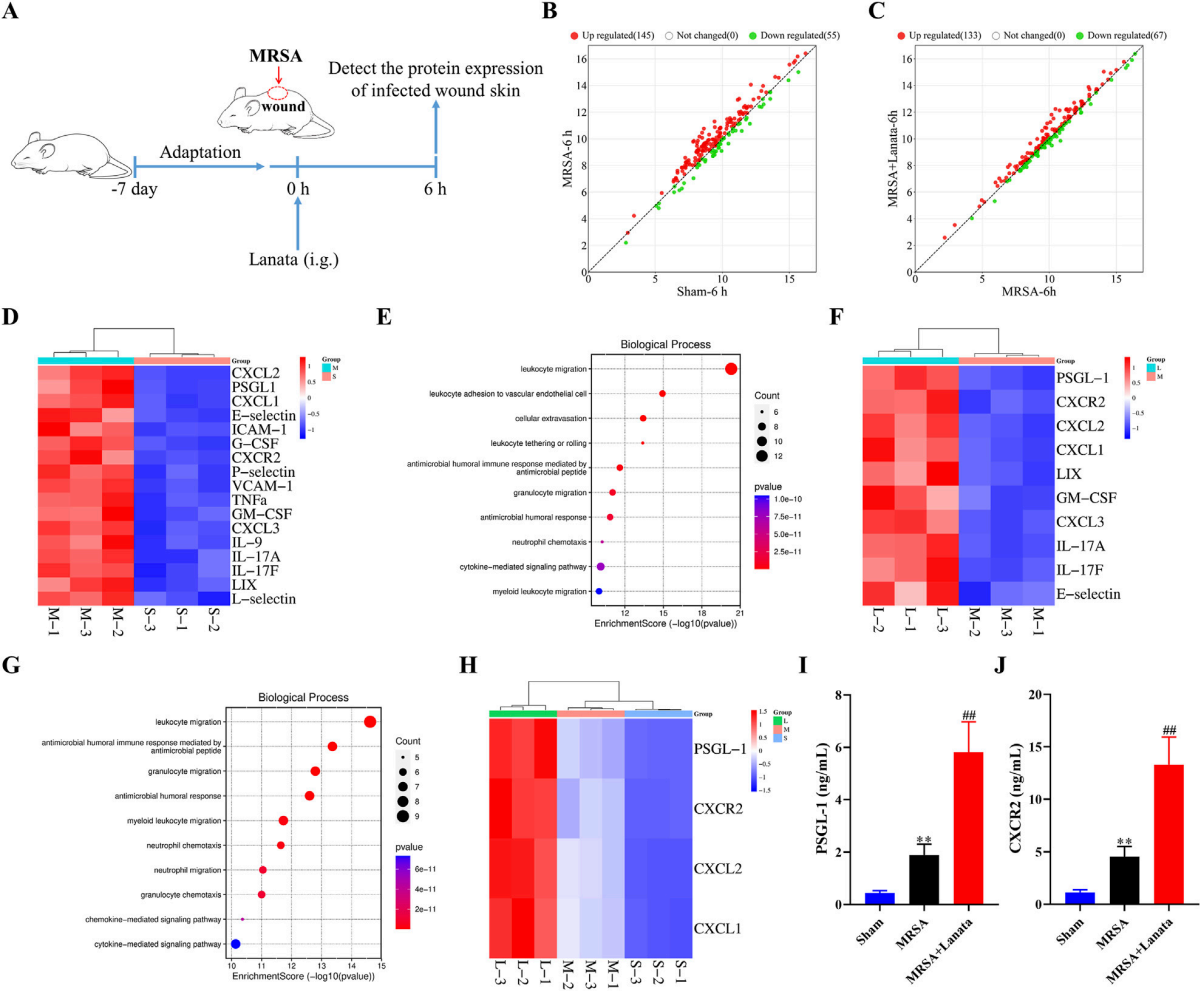
Lanata upregulated the protein levels of CXCR2 and PSGL-1 in the infected wound. **(A)** Experimental design. **(B, C)** Scatter plot of protein expression between groups. **(D)** Cluster heatmaps and **(E)** GO enrichment analysis of DEGs between the MRSA and Sham groups. **(F)** Cluster heatmaps and **(G)** GO enrichment analysis of DEGs between the MRSA and Lanata groups. **(H)** Cluster heatmaps of the major DEGs among three groups. **(I)** Protein levels of PSGL-1 and **(J)** CXCR2 in the skin were detected by ELISA. Data were presented as the mean ± SD, *n* = 3 **(A–H)**, *n* = 6 **(I, J)**. **p < 0.01 vs. Control group; ^##^ *p* < 0.01 vs. MRSA group.

To explore the mechanism by which Lanata resists MRSA infection, protein expression in the infected wound skin at 6 h post-infection was analyzed by protein chip technology (Figure 4A). Compared with the Sham group, 145 and 55 proteins were upregulated and downregulated in the MRSA group, respectively (Figure 4B). Compared with the MRSA group, 133 and 67 proteins were upregulated and downregulated in the MRSA + Lanata group, respectively (Figure 4C). As shown in Figure 4D, 17 DEPs were identified to be upregulated in the MRSA group compared with the Sham group, and GO enrichment analysis indicated that these proteins were mainly involved in neutrophil migration and leukocyte adhesion to vascular endothelial cells (Figure 4E). Meanwhile, 10 DEPs were identified to be upregulated in the MRSA + Lanata group compared with the MRSA group (Figure 4F), which were mainly involved in leukocyte and neutrophil migration (Figure 4G).

Some DEPs identified in the comparison MRSA vs. Sham overlapped with those identified in the MRSA vs. MRSA + Lanata group, with four main upregulated DEPs (PSGL-1, CXCR2, CXCL2, and CXCL1) identified as the action target of Lanata (Figure 4H). Upregulation of PSGL-1 and CXCR2 in the infected skin was confirmed by ELISA (Figures 4I, J).

### 3.4 Lanata promoted migration of neutrophils to the air pouch

**FIGURE 5:**
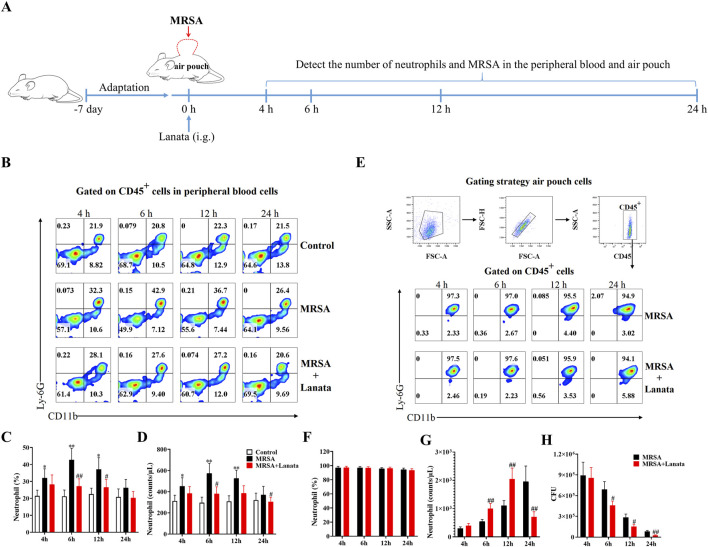
Lanata promoted migration of neutrophils to air pouch. **(A)** Experimental design. **(B, C)** Percentage and **(D)** number of neutrophils in peripheral blood leukocytes and flow cytometric analysis of CD45^+^ cells in peripheral blood leukocytes are shown in Figure 2B. **(E)** Flow cytometric analysis of neutrophils in air pouch leukocytes. **(F)** Percentage and **(G)** number of neutrophils in air pouch leukocytes. **(H)** Number of MRSA in the air pouch. Data were presented as the mean ± SD, *n* = 3. *p < 0.05, **p < 0.01 vs. Control group; ^#^ *p* < 0.05, ^##^ *p* < 0.01 vs. MRSA group.

In order to obtain the accurate number of neutrophils in the MRSA-infected site and directly study neutrophils, we established an air pouch MRSA infection mouse model. At 4 h, 6 h, 12 h, and 24 h post-infection (Figure 5A), there were almost no neutrophils migrating into the air pouch of mice in the control group (Supplementary Table S2). Compared with the MRSA group, Lanata promoted the migration of neutrophils from the peripheral blood to the air pouch (Figures 5B–G) to quickly eradicate MRSA (Figure 5H).

### 3.5 Lanata promoted activation of neutrophils in the infected air pouch

**FIGURE 6:**
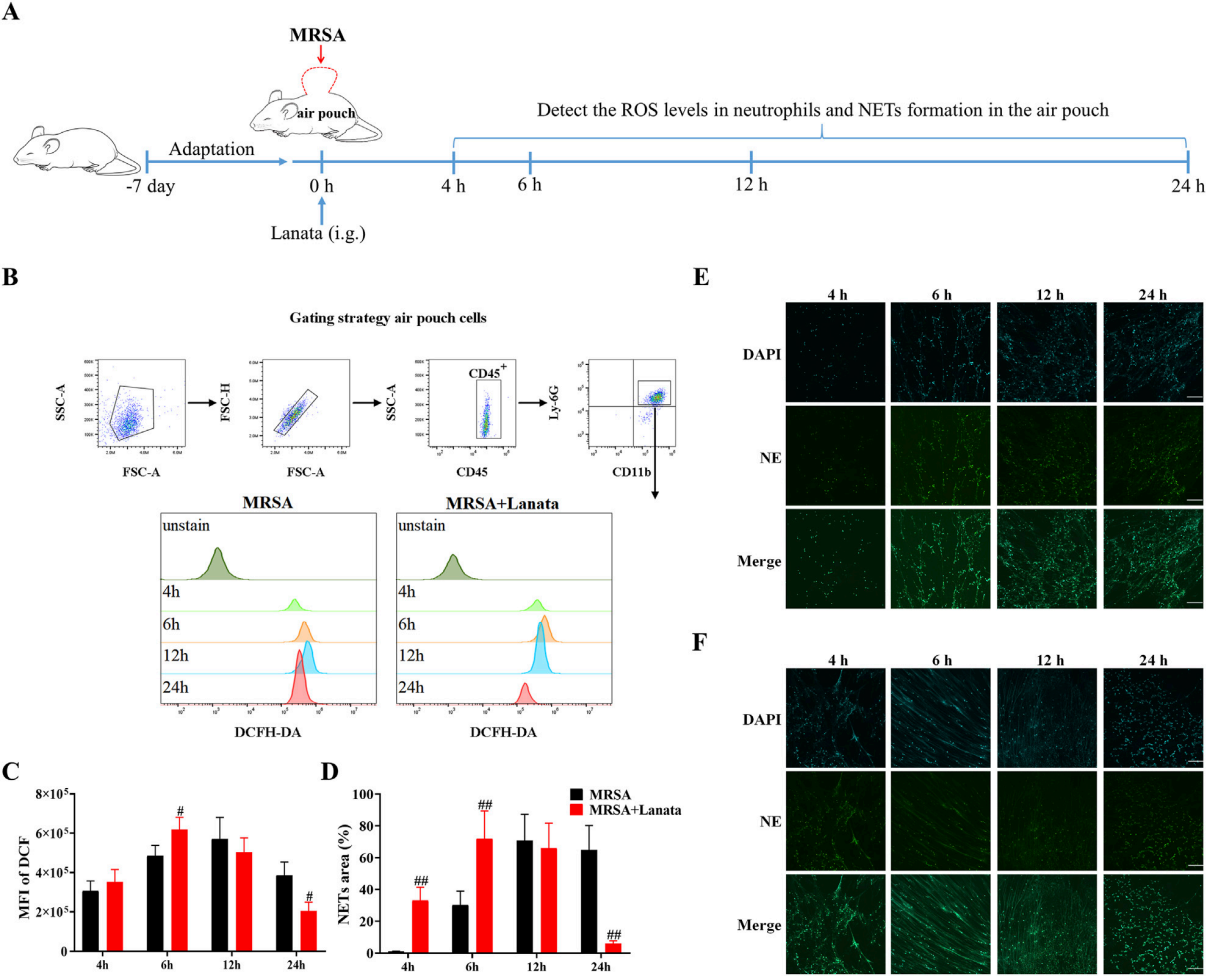
Lanata promoted activation of neutrophils in the infected air pouch. **(A)** Experimental design. **(B)** Flow cytometric analysis of the ROS level in neutrophils. The mean fluorescence intensity of DCF was used to evaluate ROS levels in neutrophils. **(C)** Mean fluorescence intensity of DCF in neutrophils. **(D)** NET area in this figure **(E, F)**. NET formation in the air pouch in **(E)** Model and **(F)** Lanata groups was observed by immunofluorescence staining. Scale bar: 100 μm. Data were presented as the mean ± SD, *n* = 3. ^#^ *p* < 0.05 vs. MRSA group.

Next, we measured the ROS levels in neutrophils and NET formation in the air pouch at 4 h, 6 h, 12 h, and 24 h post-infection to explore the effect of Lanata on activation of neutrophils (Figure 6A). As shown in Figures 6B, C, ROS levels in neutrophils in both MRSA + Lanata and MRSA groups showed the same trend of an initial increase, followed by a decrease (of note, levels in the MRSA + Lanata group were higher at 6 h and lower at 12 h post-infection than those in the MRSA group), which was related to the continuous elimination of MRSA. As shown in Figures 6D–F, Lanata promoted NET formation in the air pouch at 4 h and 6 h post-infection. At 24 h, there were almost no NETs left, which was related to the eradication of MRSA (Figure 5H).

### 3.6 Lanata regulated mRNA expression in neutrophils

**FIGURE 7:**
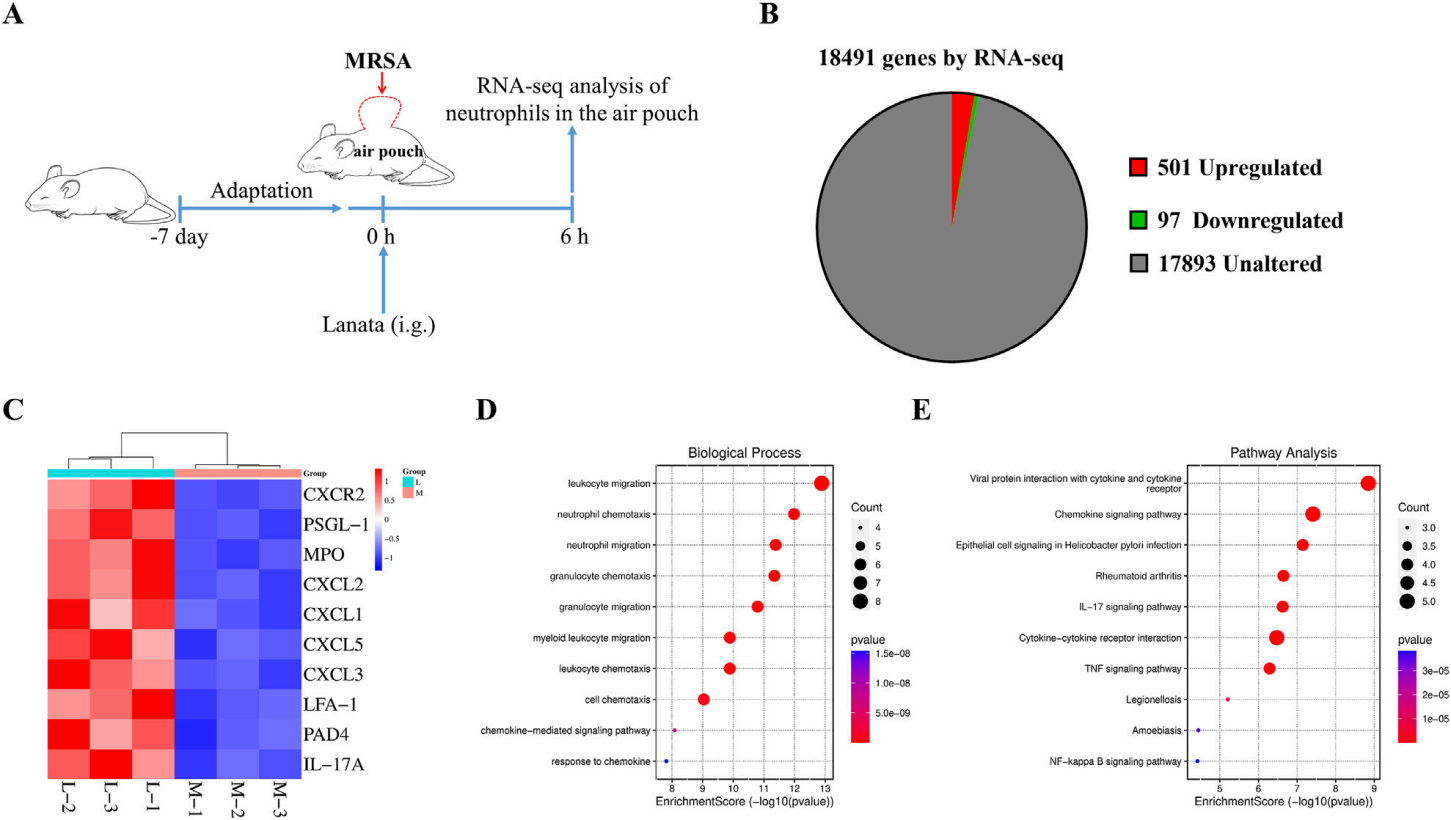
Lanata regulated mRNA expression in neutrophils. **(A)** Experimental design. **(B)** Pie chart of differentially expressed genes between the Lanata and MRSA groups. **(C)** Cluster heat map, **(D)** GO, and **(E)** KEGG enrichment analysis of DEGs between the Lanata and MRSA groups.

We conducted RNA-seq analysis on neutrophils in the air pouch at 6 h post-infection to understand the mechanism by which Lanata regulates neutrophils (Figure 7A). Compared with the MRSA group, 501 and 97 genes were significantly upregulated and downregulated in the MRSA + Lanata group, respectively (Figure 7B). The top 10 upregulated genes with Lanata included *CXCR2*, *PSGL-1*, *MPO*, *CXCL2*, *CXCL1*, *CXCL5*, *CXCL3*, *LFA-1*, *PAD4*, and *IL-17A* (Figure 7C). GO and pathway enrichment analysis indicated that the DEGs were mainly involved in neutrophil chemotaxis and migration (Figures 7D, E).

### 3.7 Lanata upregulated mRNA and protein expressions of CXCR2, PSGL-1, and MPO in neutrophils in the peripheral blood and the air pouch

**FIGURE 8:**
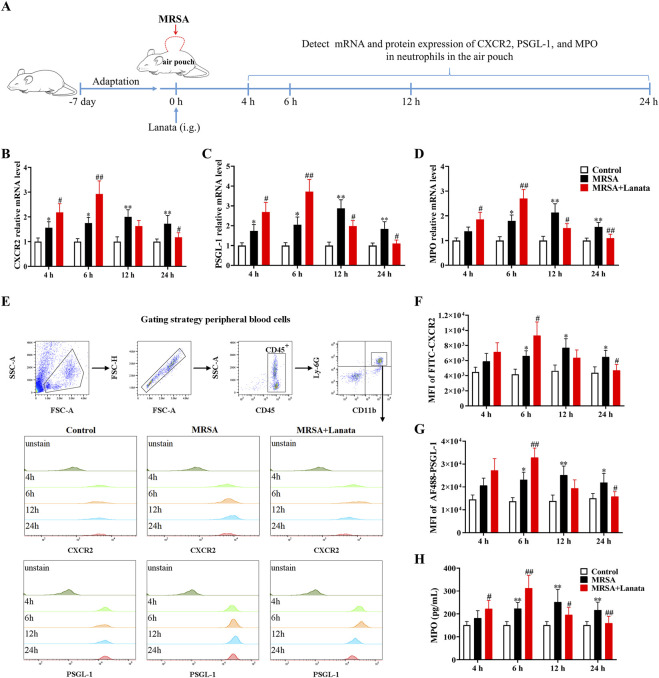
Lanata upregulated mRNA and protein expressions of CXCR2, PSGL-1, and MPO in neutrophils in peripheral blood. **(A)** Experimental design. **(B–D)** The mRNA expression levels of CXCR2, PSGL-1, and MPO in neutrophils were detected by RT-qPCR. **(E)** The protein expression of CXCR2 and MPO in neutrophils was detected by flow cytometry. Median fluorescence intensity of **(F)** CXCR2 and **(G)** PSGL-1 in neutrophils. **(H)** The concentration of MPO in neutrophils was detected by ELISA. Data were presented as the mean ± SD, *n* = 3. *p < 0.05, **p < 0.01 vs. Control group; ^#^ *p* < 0.05, ^##^ *p* < 0.01 vs. MRSA group.

To validate the RNA-seq findings, we measured the mRNA and protein expressions of CXCR2, PSGL-1, and MPO in neutrophils regulated by Lanata (Figure 8A). As shown in Figures 8B–H, mRNA and protein expression of CXCR2, PSGL-1, and MPO in neutrophils in the peripheral blood in both MRSA + Lanata and MRSA groups first increased post-infection, followed by a decrease. Of note, mRNA and protein expression of the three genes increased significantly at 6 h post-infection and were lower at 12 h in the MRSA + Lanata group compared with the MRSA group.

**FIGURE 9:**
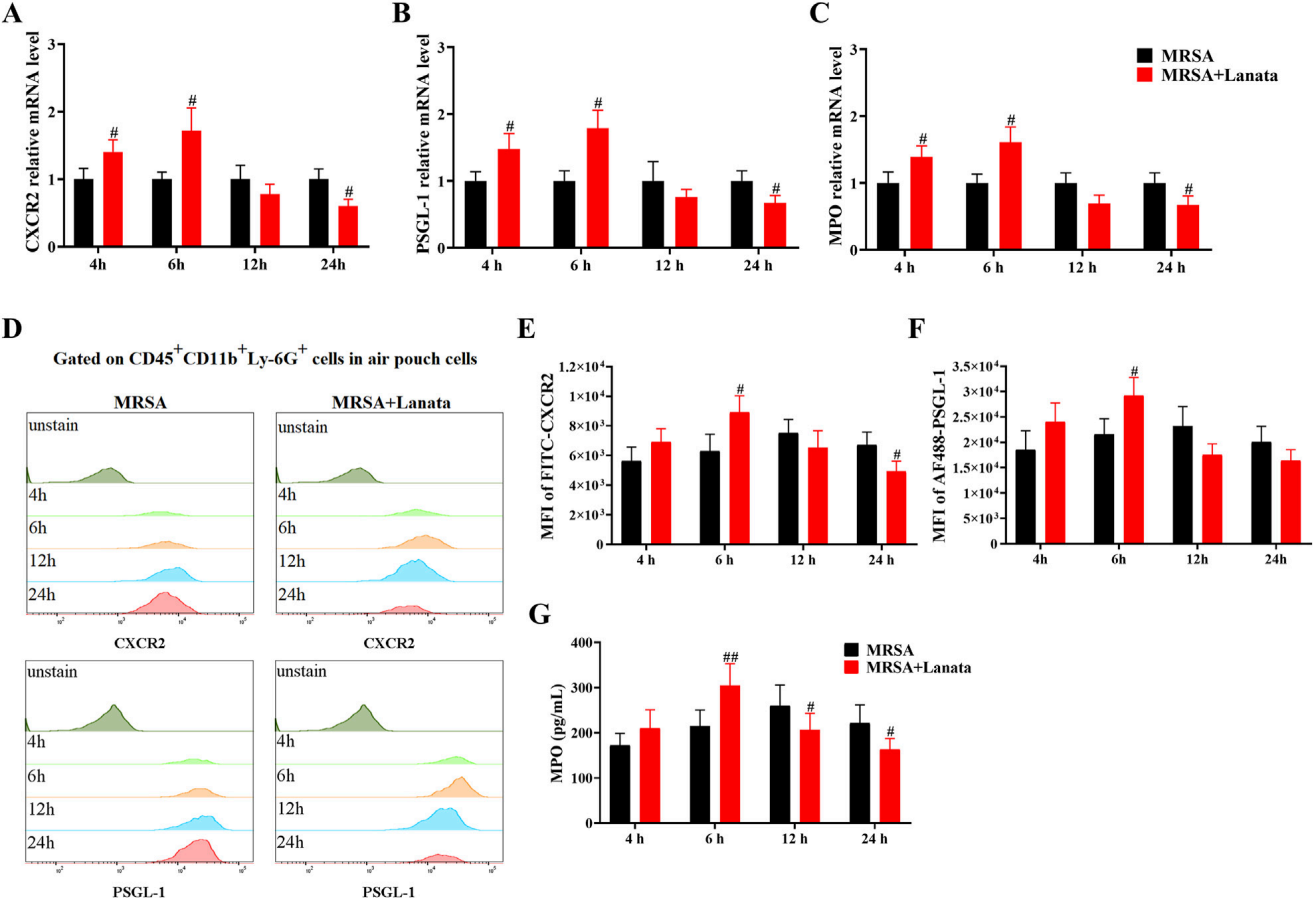
Lanata upregulated mRNA and protein expressions of CXCR2, PSGL-1, and MPO in neutrophils in the air pouch. **(A–C)** The mRNA expressions of CXCR2, PSGL-1, and MPO in neutrophils were detected by RT-qPCR. **(D)** The protein expression of CXCR2 and MPO in neutrophils was detected by flow cytometry. The median fluorescence intensity of **(E)** CXCR2 and **(F)** PSGL-1 in neutrophils. **(G)** The concentration of MPO in neutrophils was detected by ELISA. Data were presented as the mean ± SD, *n* = 3. ^#^ *p* < 0.05, ^##^ *p* < 0.01 vs. MRSA group.

Similarly, the expression of CXCR2, PSGL-1, and MPO in neutrophils in the air pouch in the MRSA + Lanata group increased significantly at 6 h post-infection and were lower than those in the MRSA group at 12 h (Figures 9A–G).

### 3.8 Inhibition of CXCR2 and MPO significantly attenuated the effect of Lanata on promoting migration and activation of neutrophils

**FIGURE 10:**
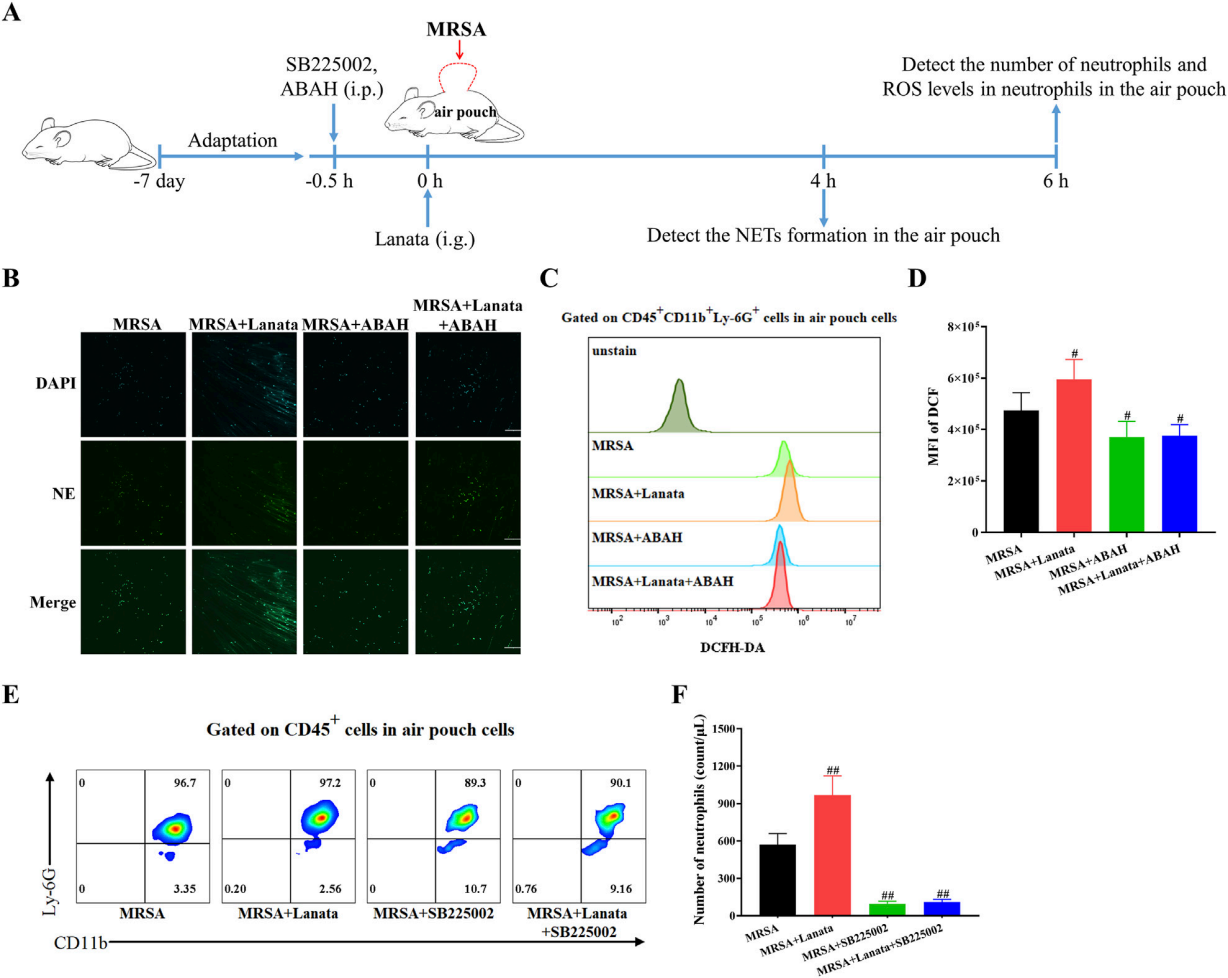
Inhibition of CXCR2 and MPO significantly attenuated the effect of Lanata on promoting migration and activation of neutrophils. **(A)** Experimental design. **(B)** Immunofluorescence staining was used to observe the NET formation in the air pouch of mice. Scale bar: 100 μm. **(C, D)** Mean fluorescence intensity of DCF in neutrophils in the air pouch of mice was detected by flow cytometry at 6 h post-infection. **(E, F)** The number of neutrophils in the air pouch was detected by flow cytometry at 6 h post-infection. Data were presented as the mean ± SD, *n* = 4. ^#^ *p* < 0.05, ^##^ *p* < 0.01 vs. MRSA group.

To further investigate the role of CXCR2 and MPO in Lanata-promoted migration and activation of neutrophils, we used ABAH and SB225002 to inhibit MPO and CXCR2, respectively (Figure 10A). As shown in Figure 10B, in the absence of treatment, there was no NET formation in the air pouch in the MRSA group at 4 h post-infection, while NETs had already begun to be released in the MRSA + Lanata group. However, after administration of ABAH, there was almost no NET formation in the MRSA + Lanata group. Similarly, in the absence of treatment, ROS levels in neutrophils in the air pouch in the MRSA + Lanata group were significantly higher than that in the MRSA group at 6 h post-infection (Figures 10C, D). However, after ABAH treatment, ROS levels in neutrophils in the two groups were significantly reduced without significant differences between groups.

As shown in Figures 10E, F, the number of neutrophils in the air pouch in the MRSA + Lanata group was significantly higher than that in the MRSA group at 6 h post-infection. However, after administration of SB225002, the number of neutrophils in the two groups was significantly reduced, and no significant differences were observed between groups.

## 4 Discussion

When tissue is infected by bacteria, a large number of neutrophils from the bone marrow are released into the peripheral blood (Heim et al., 2015) and migrate across the vascular barrier to the infected site to eradicate the bacteria. In this study, we found that after the wound was infected with MRSA and in the absence of treatment, neutrophils from the bone marrow were released into the peripheral blood and migrated to the infected wound to rapidly eradicate the bacteria, with the number of neutrophils in the wound skin reaching its peak at 24 h post-infection. Treatment with Lanata accelerated this migration and eradicated MRSA earlier, specifically in the acute stage (6 h and 12 h) of infection. Moreover, we found that the expression of PSGL-1 and CXCR2 was significantly increased at 6 h post-infection in the wound skin of Lanata-treated mice. PSGL-1 and CXCR2 are highly expressed on the surface of neutrophils and play an important role in recruiting neutrophils to migrate to inflammatory sites (Stadtmann et al., 2013; Wang et al., 2022). Therefore, our findings suggested that Lanata may have a direct effect on neutrophils by upregulating the expression of PSGL-1 and CXCR2, thereby accelerating the migration of neutrophils to the infected wounds to rapidly eradicate MRSA and promote the healing of infected wounds.

Given that the skin infection model is not suitable for the direct study of neutrophils (it is difficult to obtain neutrophils from the skin), we used the subcutaneous air pouch model–an *in vivo* model in which sterile air is injected into the back of mice to form an air pouch, where irritants can be injected to cause an inflammatory response (Fehrenbacher and McCarson, 2021). The characteristics and changes in the inflammatory response derived from MRSA infection were observed by collecting immune cells in the air pouch. Using this model, we found that Lanata promoted the migration of neutrophils from the peripheral blood to the air pouch to quickly eradicate MRSA in the acute stage of infection.

When neutrophils reach the infected site, specific receptors on their surfaces bind to the bacteria and induce the neutrophils to phagocytose the bacteria and form phagosomes within the neutrophils (Rungelrath and DeLeo, 2021). Neutrophils then eradicate bacteria by releasing ROS and NETs, which are signs of neutrophil activation (Winterbourn et al., 2016). Our results showed that ROS levels in the neutrophils in the air pouch of mice in the MRSA + Lanata group were higher than those in the MRSA group at 4 h and 6 h post-infection, reaching the peak at 6 h, suggesting that Lanata promoted the activation of neutrophils in the acute stage of infection and enhanced the bactericidal ability of neutrophils. At 12 h, ROS levels in the neutrophils of the Lanata-treated mice began to decrease, indicating a gradual decrease in neutrophil activity with the gradual eradication of MRSA, until almost complete eradication at 24 h. Overall, changes in ROS levels occurred earlier in the MRSA + Lanata group, as compared with the MRSA group (i.e., accelerated by 6 h approximately).

In addition to producing ROS, a large number of studies have shown that neutrophils can capture and eradicate bacteria by releasing NETs (Brinkmann et al., 2004). NETs are a network structure composed of nuclear chromatin; histone; granular proteins such as NE, MPO, or cathepsin G; and cytoplasmic proteins such as glycolytic enzymes and catalase (Urban et al., 2009; Schultz et al., 2022). When neutrophils are activated, they undergo through a series of processes to produce NETs (Cristinziano et al., 2022). NETs are released to the extracellular space, greatly increasing the contact area with the bacteria, and as a consequence, the bacteria are dissociated by the granular proteins and histones of the NETs. We found that NET formation and release to the air pouch was accelerated in the MRSA + Lanata group during the acute stage of the infection (at 4 h and 6 h), while there were almost no NETs left in the air pouch of Lanata-treated mice at 24 h compared with the MRSA group. Our results suggest that Lanata increased the speed and intensity of NET release in the acute stage of infection to quickly eradicate MRSA.

Next, we conducted RNA-seq analysis on the neutrophils in the air pouch at 6 h post-infection to understand the mechanism by which Lanata regulates neutrophils. We found that Lanata significantly upregulated the expressions of CXCR2, PSGL-1, and MPO mRNA in neutrophils. Using RT-qPCR, flow cytometry, and ELISA, we found that the mRNA and protein levels of CXCR2, PSGL-1, and MPO in the neutrophils of the MRSA + Lanata group increased significantly at 6 h post-infection and were lower than those in the MRSA group after 12 h.

It has been described that upon infection, neutrophils in the peripheral blood need to go through a series of processes to migrate to the local tissue. In the first step of this process, neutrophils must contact with the vascular endothelial cells at the site of infection for neutrophils to leave the blood stream (Eriksson et al., 2001; Sperandio et al., 2003). Endothelial cells are activated by inflammatory cytokines released by sentinel cells in the infected tissues, which induce the rapid expression of P-selectin and E-selectin on their surfaces (Sais et al., 1997; Liu et al., 2010). PSGL-1 is the most effective selectin ligand expressed on the surface of neutrophils (Xia et al., 2002). When PSGL-1 of the neutrophils present in the blood stream binds to P-selectin and E-selectin of endothelial cells, the neutrophils are captured on the surface of the vascular endothelium. A previous study has shown that after PSGL-1 was knocked out, the number of neutrophils in inflamed sites of mice decreased significantly (Zhang et al., 2020), suggesting that this interaction driven by PSGL-1 is essential for neutrophil migration to inflamed sites. In our study, we found that PSGL-1 expression in the neutrophils in the peripheral blood of Lanata-treated mice was increased in the acute stage of infection, improving the efficiency of neutrophils being captured by endothelial cells and allowing that neutrophils in the blood stream to be quickly captured by the vascular endothelium for subsequent migration. This may explain the significant increase in the number of neutrophils in the air pouch in the MRSA + Lanata group when compared with the MRSA group.

When neutrophils in the peripheral blood are captured by the vascular endothelium, they migrate to the inflamed site influenced by the concentration of chemokines such as CXCL1 and CXCL2 through CXCR2 (Michael and Vermeren, 2019). A previous study showed that inhibition of CXCR2 significantly attenuated the migration of neutrophils to inflammatory sites (Wu et al., 2020). In our study, we found that CXCR2 expression in neutrophils in peripheral blood in the Lanata group followed the same trend as PSGL-1 (i.e., increase in the acute stage of infection as compared with the MRSA group). This greatly improved the chemotaxis and migration ability of the neutrophils and promoted the rapid migration of neutrophils in peripheral blood to the infected site. After treatment with the CXCR2 inhibitor SB225002, the number of neutrophils in both MRSA + Lanata and MRSA groups was significantly reduced, and no significant differences were observed between both groups, suggesting that inhibition of CXCR2 significantly attenuated the effect of Lanata on promoting migration of neutrophils.

MPO is a heme protein that is abundantly stored in the neutrophils’ azurophilic granules (Klebanoff et al., 2013). In phagosomes, MPO catalyzes the reaction of hydrogen peroxide and chloride to produce ROS-hypochlorous acid, which has a potent bactericidal ability (Rigby and DeLeo, 2012). Previous studies have shown that MPO plays an important role not only in the production of ROS in neutrophils but also in the release of NETs (Papayannopoulos et al., 2010). We found that, similar to that observed with expression of PSGL-1 and CXCR2, MPO expression in the neutrophils of Lanata-treated mice increased at 4 h and 6 h post-infection, reaching the peak at 6 h. As a consequence, this not only increased ROS levels in the neutrophils but also promoted the release of NETs. Upon treatment with the MPO inhibitor ABAH, ROS levels in the neutrophils in both the MRSA + Lanata and MRSA groups were significantly reduced with no significant differences between the two groups, and the release of NETs was delayed. Together, our results suggest that inhibition of MPO significantly attenuated the effect of Lanata on promoting activation of neutrophils.

Our study demonstrated that mice treated with Lanata, which is called the “the holy medicine of pustule,” resists MRSA infection by promoting migration and activation of neutrophils. This finding provides a scientific basis for the use of Lanata for the treatment of skin infections, as well as a new option for the treatment of infectious diseases. Future research including *in vitro* mechanistic assays aimed to uncover the direct effect of Lanata on neutrophils is warranted.

## 5 Conclusion

Our study reveals the mechanism by which mice treated with *Panzerina lanata* resists MRSA infection by promoting migration and activation of neutrophils. Under the action of *Panzerina lanata*, the captured speed of neutrophils with high expression of PSGL-1 in peripheral blood by vascular endothelial cells was greatly improved, and the neutrophils with high expression of CXCR2 acquired stronger chemotaxis and migration ability; consequently, a larger number of neutrophils arrived to the infected site; upon reaching the infected site, the neutrophils with high expression of MPO were activated after recognizing MRSA, further strengthening production of ROS and rapid release of NETs to eradicate MRSA, thus exerting an anti-infection effect.

## Data Availability

The transcriptome sequencing data presented in the study are deposited in the NCBI SRA BioProject repository, accession number PRJNA1163875.

## Abbreviations

ABAH, 4-aminobenzoic hydrazide; CXCR2, CXC chemokine receptor 2; DEGs, differentially expressed genes; DEPs, differentially expressed proteins; H&E, hematoxylin and eosin; MFI, mean fluorescence intensity; MPO, myeloperoxidase; MRSA, methicillin-resistant *Staphylococcus aureus*; NE, neutrophil elastase; NETs, neutrophil extracellular traps; NOX, nicotinamide adenine dinucleotide phosphate oxidase; PAD4, peptidyl arginine deiminase 4; PSGL-1, P-selectin glycoprotein ligand-1; RBC, red blood cell; ROS, reactive oxygen species; TTC, 2,3,5-triphenyltetrazolium chloride.
