# Supplementary material for: Panzerina lanata accelerates methicillin-resistant Staphylococcus aureus eradication by promoting migration and activation of neutrophils
Source: Front Pharmacol. 2025 Jan 14;15:1501744. doi: 10.3389/fphar.2024.1501744 (PMC11772359; doi:10.3389/fphar.2024.1501744)
Supplement: Supplementary file 1 [file Table1.docx]

Supplementary Material

**Supplementary Table 1 Primer sequences for qRT-PCR**

| Gene | Sequences (5’ - 3’) |
| --- | --- |
| CXCR2-F | CCCTCTTTAAGGCCCACATG |
| CXCR2-R | AAGGACGACAGCGAAGATGAC |
| PSGL-1-F | GAAAGGGCTGATTGTGACCCC |
| PSGL-1-R | AGTAGTTCCGCACTGGGTACA |
| MPO-F | AGTTGTGCTGAGCTGTATGGA |
| MPO-R | CGGCTGCTTGAAGTAAAACAGG |
| GAPDH-F | CATGGCCTTCCGTGTTCCTA |
| GAPDH-R | GCGGCACGTCAGATCCA |

**Supplementary Table 2 Number of neutrophils in the air pouch of mice in each group at different time points (counts/μL)**

| Group | 4 h | 6 h | 12 h | 24 h |
| --- | --- | --- | --- | --- |
| Control | 2±1 | 3±1 | 5±2 | 3±1 |
| MRSA | 302±52** | 545±75** | 1108±174** | 1954±553** |
| MRSA+Lanata | 400±73 | 998±184**^##^** | 2048±386**^##^** | 707±183**^##^** |

Note: Data were presented as the mean±SD, n=6. ***p* < 0.01 *vs* Control; ^##^*p* < 0.01 *vs* MRSA
